# Valuing patient perspectives in the context of eating disorders

**DOI:** 10.1007/s40519-023-01635-3

**Published:** 2024-02-04

**Authors:** Harshita H. Jaiprakash, Amy MacKinnon, Sarah Arnaud, Jacob P. Neal

**Affiliations:** 1 Department of Philosophy and the Rotman Institute of Philosophy, Western University, London, ON Canada; 2 Department of Philosophy, Clemson University, Clemson, SC USA

**Keywords:** Anorexia nervosa, Patient values, DSM-V, Acceptance and commitment therapy, Patient integration

## Abstract

**Purpose:**

This paper advocates for the inclusion of patient perspectives in the diagnosis and treatment of eating disorders (EDs) for ethical, epistemological, and pragmatic reasons. We build upon the ideas of a recent editorial published in this journal. Using EDs as their example, the authors argue against dominant DSM-oriented approaches in favor of an increased focus on understanding patients’ subjective experiences. We argue that their analysis stops too soon for the development of practical—and actionable—insights into how to effect the integration of first-person and third-person accounts of EDs.

**Methods:**

Contextual analysis was used to make the case for patient perspectives.

**Results:**

We use anorexia nervosa (AN) as an example to demonstrate how the integration of patient manifestations and voices offers a promising methodology to improve patient diagnosis and treatment. We suggest that Acceptance and Commitment Therapy (ACT) can support patients with AN by reconciling their values with the values that arise from a clinician’s duty of care.

**Conclusions:**

We conclude that there are no good scientific reasons to exclude first-person perspectives of EDs in psychiatry.

Level of evidence: *Level V: Opinions based on clinical experience*.

## Introduction

In their recent editorial, Stanghellini and colleagues [1] argue against dominant DSM-oriented approaches, instead advocating for an increased focus on understanding patients and their subjective experiences. Taking EDs as their case study, they develop three critiques of current psychiatric practice, arguing that a DSM-focused approach is destined to fail [1]. We agree with the broad outlines of their critique; however, their analysis stops too soon and thus prevents them from developing practical—and actionable—insights into how to effect the integration of first-person and third-person accounts of EDs. In this paper, we go one step further. We advocate for the inclusion of patient perspectives, not only for epistemological reasons, but also for ethical and pragmatic ones. We suggest an account of normativity for the treatment of AN that avoids the pitfalls of paternalism, and we present ACT as a more practical solution to the problem of conflicting values.

### The DSM and its discontents: theory and observation in psychiatric nosology

According to most philosophers, empirical observations are theory-laden, since any observation is interpreted in light of the previous theoretical and conceptual commitments of the observer. Ironically, in the case of psychiatry, it is the putatively theory-free DSM criteria themselves that provide the lens through which psychiatrists see a patient’s symptoms. For example, since AN is classified as an eating disorder, the DSM criteria directs the psychiatrist to focus primarily on eating behaviors. This, however, diverts attention from other relevant factors that may be underlying such behaviors (e.g., desire for control) [2]. The psychiatrist’s reliance on the DSM influences her observation of the patient since it makes certain behaviors appear more clinically salient than others.

The problems of operationalization identified by Stanghellini and colleagues [1] are not unique to psychiatry. Scientific observation is always constrained, and operationalization is widespread; yet, we do not see similar concerns raised in biomedicine about the risks of tunnel vision, shoehorning, or the lack of “purely empirical” observations that are present in psychiatry. Why are these critiques against the use of the DSM in psychiatry so compelling?

The answer to this puzzle has to do with both the status of the DSM as a diagnostic tool as well as the current state of psychiatric knowledge. The problem with the DSM cannot be that it influences clinical observations, but rather that its diagnostic criteria influence clinical observations *in pernicious ways*. Critics claim that one way this influence manifests is through the DSM’s failure to pick out natural kinds or even particularly useful psychiatric kinds. Kendler [3] argues that current psychiatric categories are at best “working hypotheses,” and we should expect them to change as psychiatry matures. Thus, it is not unreasonable to assume that the diagnostic criteria for many mental disorders currently include ineffective criteria while at the same time omit certain central features of these disorders. It is when we see the DSM as a flawed diagnostic and research tool that the tunnel vision it engenders becomes a problem.

### Integration of first-person perspectives

Like us, Stanghellini and colleagues [1] also argue for the inclusion of first-person perspectives in psychiatric practice and the development of psychiatric nosology. But what does it mean for psychiatry to include patients’ perspectives? This term can take on two distinct meanings, one that refers to the phenomenological experiences or manifestation of the disorder, and the other that refers to first-person testimonies about the disorder. When advocating for the inclusion of first-person perspectives, some researchers refer to patients’ phenomenological *manifestations* or experiences*,* such as perceptual factors [2] or subjective thoughts and feelings [4, 5]*.* Others refer to patients’ *voices* and *self-reports,* arguing that these should be taken into account, especially when patient voices contrast with clinical accounts [6].

Since the DSM was intentionally constructed to exclude subjective experiences, it lacks manifestation integration. This is evident in the current diagnostic criteria for AN, which focuses on observable behaviors such as food intake and body weight rather than the diversity of first-person feelings, beliefs, and thoughts. Voice-integration is also lacking. Diagnostic manuals aim for reliability to make these diagnostic tools practical. However, reliability often arises at the expense of validity [7], leading to diagnostic criteria that overlook patients’ concerns [1]. The potential invalidity and ineffectiveness of diagnostic tools arise from the methods by which we build nosography, i.e., clinical assessments through checklists, instead of patients’ testimonies and perspectives.

The lack of integration of patient perspectives is problematic for multiple pragmatic reasons. AN patients frequently relapse after weight normalization [8], and this is partially a sign of treatment inefficacy due to an incomplete understanding of the symptomatology. Because emotional aspects of AN have historically been excluded from diagnosis, characterization, and treatment considerations, they offer untapped potential for improving our understanding and treatment of AN. If we were to engage in a more serious study of these components, it is possible that we could identify reliable *and* valid manifestation symptoms.

While necessary, the inclusion of patient voices and manifestations into the current diagnostic criteria faces important challenges. The first set of challenges is *political*: the DSM task force refused to integrate patients into work groups for the DSM-5, threatened by the subjectivity of patients' reports and how it conflicts with psychiatry's aim to be recognized as an objective science. The second set of challenges concerns the *methods* of integration, or how to gather and translate the experiential components for inclusion in the descriptions of AN. Another methodological challenge is about conflicting inter- and intrapersonal reports. AN patients can have some differing feelings depending on their life trajectories and experiences. This variation can occur from one patient to another, or can occur as conflicting desires within a patient him or herself (e.g., restricting food consumption vs. remaining healthy). This opens the door to the questions of values that Stanghellini and colleagues [1] introduce in their discussion of normativity in psychiatry.

### Normativity without paternalism

Value conflicts may arise between clinicians and patients with AN because the egosyntonic nature of AN causes patients to identify with their disorder. Thus, a patient might see their behaviors, e.g., restricting caloric intake or exercising excessively, not as disordered but as reasonable and in alignment with their values. If patient values are not understood, clinicians will lack the necessary groundwork to communicate effectively and work together on a treatment plan.

There are at least two possible ways to overcome the “therapeutic collision” that arises when clinician and patient values conflict: (1) moralistic paternalism, where societal or clinical values are accepted as governing and correct, or (2) value pluralism, where we acknowledge all values as acceptable. The former solves the conflict by deferring to clinician values: if a patient values thinness at the expense of health, then clinicians would paternalistically aim to replace their values for health over the patient’s value for thinness. This could lead to force-feeding interventions, which aligns with clinician’s values, and improves the health of the patient, but clearly violates patient autonomy. Stanghellini and colleagues [1] identify the risk of this sort of moralistic paternalism, but do not discuss the extent of the problem or offer compelling ways forward. The obvious ethical concerns with this approach, however, leads to endorsing the latter approach: value pluralism.

Value pluralism, however, can be interpreted as “anything goes.” Yet, given the ethical codes and legal constraints of a clinician’s duty of care, this type of value pluralism cannot reconcile clinician values with patient values that are deemed harmful. How, then, can value pluralism be endorsed and practiced so that therapeutic interventions for AN do not eliminate a patient's values but instead help to reweight those values so that the harmful effects of the symptom-related behaviors are minimized.

We suggest that Acceptance and Commitment Therapy (ACT), which continues to be experimental in EDs, can be integrated into clinical contexts to help clinicians work with patient values. Given that patients with AN have notoriously rigid values, ACT is patient-focused and centered on considering how values can be interpreted and practiced in constructive ways. The concept of *psychological flexibility* endorsed by ACT refers to the cognitive ability to recognize that multiple potential beliefs can be associated with any given value [9]. This enables patients to choose what is best suited for their therapeutic goals—by seeing health as a value meaning having sustainable energy and not as thinness. For psychiatric disorders, such as AN, empirical research has shown that higher psychological flexibility correlates to lower risk of psychiatric disorder [10].

To help select the belief–action pair that best meets mutual clinical–patient values, ACT’s methodology focuses on *workability*. What determines workability in the therapeutic context of ACT are belief–action pairs that improve one’s health and well-being as guided by the clinician’s duty of care. Patient values are accepted a priori as valid by clinicians, and the therapeutic intervention entails supporting patients’ ability to discern and utilize the best suited beliefs and actions. Patient values then become flexible and less fixed which, in turn, helps overcome radical value pluralism while steering clear of paternalistic moralism [9]. We contend that ACT’s integration of patient voices leads to indispensable clinical awareness and validation of patient values for those with AN, consequently overcoming the problem of conflicting values.

## Discussion

This paper has taken Stanghellini and colleagues’ [1] criticisms against the current psychiatric practice one step further. We argued that there are no good scientific reasons to exclude first-person perspectives of mental disorders and that psychiatry ought to record first-person perspectives along with third-person observations. However, we identified certain challenges that must be overcome to integrate patient perspectives. We used AN as an example to demonstrate how the integration of patient manifestations and voices offers a promising methodology to improve patient diagnosis and treatment. Given that clinicians have a duty of care toward their patients which entails the values of health and well-being, considering how patients with AN interact with such values can reveal how clinical treatments play out. We argued that ACT could validate patient values while guiding them toward mutual clinician–patient goals. We hope these suggestions for including patient perspectives can further psychiatric study in both ethical and pragmatic ways.

*Strengths and limits* This paper’s strength lies in its critical insight on the theoretical underpinning of the DSM, the role of patient perspectives in the context of eating disorder nosology and treatment, along with its suggestions for applied therapeutic change when considering patient values. A limitation, however, is the lack of engagement with clinical trials which could reveal further challenges that were not explored.

*What this study adds* This paper adds to, and refines, the suggestions made by Stanghellini and colleagues, and seeks to clarify explanations about the role of patient perspectives in EDs. A unique contribution is the presentation of ACT as an approach that is centered on valuing patient perspectives as a part of therapeutic intervention which is gaining traction in applied treatment for AN and ED. This research has epistemological, ethical, and pragmatic implications in the fields of patient care, research, and clinical practice.

## Data Availability

Not applicable.
